# VOICES FROM THE MARGINS: EXPLORING INEQUITIES IN WORK AND VOLUNTEERING AMONG OLDER ADULTS

**DOI:** 10.1093/geroni/igae098.1851

**Published:** 2024-12-31

**Authors:** Christina Matz, Byeongju Ryu, Julie Miller

**Affiliations:** Chair; Co-Chair; Discussant

## Abstract

All people should have the right to choice and opportunity when it comes to engagement in paid and unpaid forms of work across the life course and particularly in later life. It is often through such roles/aspects of our identity that we derive a sense of meaning and purpose, which is critical to quality of life. We also know, however, that race, gender, birthplace, social and economic class, and additional layers of being “othered” impact the choices and opportunities available, as well as the extent to which positive (or negative) outcomes are derived. Research on paid and unpaid work in later life has not always captured the variety and complexity of lived experiences in later life. Further, statistics often obscure the most important information: how the most marginalized older workers are faring.

This symposium will explore some of these inequities in an effort to inform solutions. The first paper is a scoping review that assesses the extent to which volunteerism among economically marginalized older adults has been examined in studies. The second paper explores how older self-employed workers’ finances and health were impacted by COVID-19. The third paper considers gender disparities in wages among older workers in association with occupational sex segregation. The final paper examines the ethnoracial differences in formal and informal volunteering among individuals age 50 and older. A discussant will reflect on these studies and the need for continued research that considers intersectionality in opportunities and experiences for paid and unpaid work in later life. Aging Workforce Interest Group Sponsored Symposium

